# FOXO1 contributes to diabetic cardiomyopathy via inducing imbalanced oxidative metabolism in type 1 diabetes

**DOI:** 10.1111/jcmm.15418

**Published:** 2020-05-25

**Authors:** Dan Yan, Yin Cai, Jierong Luo, Jingjin Liu, Xia Li, Fan Ying, Xiang Xie, Aimin Xu, Xiaosong Ma, Zhengyuan Xia

**Affiliations:** ^1^ Department of Anesthesiology The University of Hong Kong Hong Kong China; ^2^ Diabetes Center Shenzhen University Shenzhen China; ^3^ Department of Anesthesiology Institute of Anesthesiology and Critical Care Medicine Union Hospital Tongji Medical College Huazhong University of Science and Technology Wuhan China; ^4^ Department of Medicine The University of Hong Kong Hong Kong China

**Keywords:** diabetic cardiomyopathy, FOXO1, oxidative metabolism

## Abstract

Forkhead box protein O1 (FOXO1), a nuclear transcription factor, is preferably activated in the myocardium of diabetic mice. However, its role and mechanism in the development of diabetic cardiomyopathy in non‐obese insulin‐deficient diabetes are unclear. We hypothesized that cardiac FOXO1 over‐activation was attributable to the imbalanced myocardial oxidative metabolism and mitochondrial and cardiac dysfunction in type 1 diabetes. FOXO1‐selective inhibitor AS1842856 was administered to streptozotocin‐induced diabetic (D) rats, and cardiac functions, mitochondrial enzymes PDK4 and CPT1 and mitochondrial function were assessed. Primary cardiomyocytes isolated from non‐diabetic control (C) and D rats were treated with or without 1 µM AS1842856 and underwent Seahorse experiment to determine the effects of glucose, palmitate and pyruvate on cardiomyocyte bioenergetics. The results showed diabetic hearts displayed elevated FOXO1 nuclear translocation, concomitant with cardiac and mitochondrial dysfunction (manifested as elevated mtROS level and reduced mitochondrial membrane potential) and increased cell apoptosis (all *P* < .05, D vs C). Diabetic myocardium showed impaired glycolysis, glucose oxidation and elevated fatty acid oxidation and enhanced PDK4 and CPT1 expression. AS1842856 attenuated or prevented all these changes except for glycolysis. We concluded that FOXO1 activation, through stimulating PDK4 and CPT1, shifts substrate selection from glucose to fatty acid and causes mitochondrial and cardiac dysfunction.

## 1 INTRODUCTION

Diabetic cardiomyopathy (DCM) is defined as a clinical presentation of ventricular dysfunction caused by alterations in cardiac energy substrates, the occurrence of which is independent of arterial and structural heart diseases.^1^, ^2^ Type 1 diabetes mellitus (T1DM) patients with poor glycaemic control still have a high propensity to develop DCM^3^ and eventually cause heart failure.^4^ A shift of fuel preference that resembles type 2 diabetes mellitus (T2DM) was observed in T1DM patients.^5^ Impaired glucose oxidation in hearts of T1DM is partially attributable to diminished cardiac insulin signalling and thus imposes the heart more reliable on fatty acid β‐oxidation for energy supply.^6^ The resulted imbalanced oxidative metabolism, manifested by the shift from glucose oxidation to fatty acid oxidation, is proposed to be an underlying cause of mitochondrial dysfunction and often associated with cardiac dysfunction.^7^ Nevertheless, the initial mechanism that triggers the imbalanced oxidative metabolism in type 1 diabetic heart is still unknown.

Forkhead box protein O1 (FOXO1) is a member of ‘O’ subgroup of Forkhead box family of transcriptional factors.^8^, ^9^ Accumulating evidence has demonstrated the role of FOXO1 in the regulation of cardiac metabolism.^10^, ^11^, ^12^ FOXO1 decreases glucose oxidation rate through enhancing pyruvate dehydrogenase kinase 4 (PDK4) expression and thus impairs right ventricular function in pulmonary hypertension in rats.^13^ Moreover, in primary cardiomyocytes, activation of FOXO1 promotes CD36 (fatty acid transporter) translocation to the membrane, which in turn facilitates fatty acid uptake.^11^ These observations suggest a crucial role of FOXO1 in modulating glucose and fatty acid metabolism in the heart. Intriguingly, FOXO1 has been demonstrated to be over‐activated in heart from high‐fat diet–induced T2DM mice.^12^ And, the aberrant activated FOXO1 boosts morphological and functional myocardial alterations that resemble DCM by mediating cardiac insulin resistance and inducing lipid accumulation and lipotoxicity.^12^ As the insulin action in T1DM patients differs from that in T2DM, the important questions remaining unsolved are whether or not FOXO1 is activated in non‐obese insulin‐deficient diabetes and whether activation of FOXO1 contributes to cardiac metabolic flexibility with the switch from glucose oxidation to fatty acid oxidation in the myocardium of T1DM.

The imbalanced oxidative metabolism, especially increment of fatty acid oxidation, induces mitochondrial reactive oxygen species (mtROS) production,^14^ resulting in mitochondrial dysfunction and cell apoptosis,^7^ which may exacerbate DCM. Thus, how FOXO1 activation affects mtROS and cardiac myocyte apoptosis and eventually leads to DCM in T1DM is also needed to be elucidated. Considering the prospect for clinical translation, FOXO1‐selective inhibitor, AS1842856 (AS), was administered to streptozotocin (STZ)‐induced diabetic (D) rats as well as the isolated primary diabetic cardiomyocytes to directly assess the role of FOXO1 activation in the imbalanced oxidative metabolism and, in the meanwhile, to study the effects of AS on mitochondrial dysfunction and impaired cardiac function in STZ‐induced diabetic hearts.

## 2 MATERIALS AND METHODS

### 2.1 Animal treatment

Male Sprague Dawley rats (200 ~ 250 g) obtained from the Laboratory Animal Unit (The University of Hong Kong) were used in this study, and animal protocols were reviewed and approved by the Committee on the Use of Live Animals in Teaching and Research (CULATR) in the University of Hong Kong. As described,^15^ type 1 diabetes was induced by a single tail vein injection of STZ (Sigma‐Aldrich) in 0.1 M citrate buffer (pH 4.5) or citrate buffer alone as control at 65 mg/kg bodyweight. After 72 hours of injection, rats with blood glucose over 16.7 mm/L were considered as having diabetes. At fourth week of diabetes induction, diabetic rats were administered AS (Glixx Lab) at the dosage of 50 mg/kg bodyweight (BW) by gavage twice a day for the following one week. Before administered to animals, the drug AS1842856 was dissolved in 10% w/v β‐cyclodextrin and underwent ultrasound homogenization until obtaining homogenous suspension liquid. Our preliminary and previous study demonstrated that STZ‐induced diabetic rats at the early stage of the disease (4 weeks) developed diabetic cardiomyopathy, manifested as myocyte apoptosis, hypertrophy and cardiac dysfunction.^16^ Therefore, we determined to apply pharmacological intervention starting from the fourth week of diabetes induction. At the end of the experiment, rats underwent cardiac function evaluation or were killed for heart tissue collection.

### 2.2 Pressure‐volume analysis of left ventricular function

Pressure‐volume analysis was conducted as our previous report.^17^ To be brief, rats were anaesthetized by intraperitoneal injection of ketamine (100 mg/kg BW) and xylazine (10 mg/kg BW). Trachea was intubated with a cannula connected to a rodent ventilator (Harvard Rodent Ventilator Model 683) with a tidal volume of 1.0 mL/100 g bodyweight (75 breaths/min). Then, the catheter was inserted to beating heart through right carotid artery and the conductance catheter was connected to a computer equipped with an advantage PV control box software (AD Instruments). The baseline cardiac functional parameters were recorded, including HR, SV, stroke work (SW), cardiac output (CO), left ventricular end‐diastolic pressure (LVEDP), left ventricular end‐systolic pressure (LVESP), maximal slope of systolic pressure increment (dP/dt max), diastolic decrement (dP/dt min) and the relaxation time constant calculated by Weiss method (Tau).

### 2.3 Extraction of total RNA and quantitative real‐time polymerase chain reaction analysis

Extraction of cardiac total RNA and quantitative real‐time PCR were performed as before.^18^ Gene‐specific primers were as follows: rat FOXO1 forward: 5′‐CAGCAAATCAAGTTATGGAGGA‐3′, reverse: 5′‐TATCATTGTGGGGAGGAGAGTC‐3′; rat PDK4 forward: 5′‐TCCTTCACACCTTCACCACA‐3′, reverse: 5′‐AAAGAGGCGGTCAGTAATCC‐3′; and rat GAPDH forward: 5′‐GGGTGTGAACCACGAGAAAT‐3′, reverse: 5′‐ACTGTGGTCATGAGCCCTTC‐3′.

### 2.4 Primary cardiomyocyte isolation and culture

As reported,^19^ rat hearts were immediately removed and intubated with a cannula to the Langendorff System. Perfuse heart with perfusion buffer [120.4 mM NaCl, 14.7 mM KCl, 0.6 mM KH_2_PO_4_, 0.6 mM Na_2_HPO_4_, 1.2 mM MgSO_4_⋅7H_2_O, 10 mM Na‐HEPES, 4.6 mM NaHCO_3_, 30 mM taurine, 10 mM 2,3‐butanedione monoxime (BDM), 5.5 mM glucose (pH 7.1)] until the blood in heart was washed out, and then, perfuse heart with digestion buffer containing 1.5 mg/mL collagenase II. After full digestion, the heart was then gently eased apart in the stopping solution containing 10% calf serum and 12.5 µM CaCl_2_. The resulted tissue pieces were filtrated through a 70‐μm nylon mesh to obtain isolated cells. Then, the isolated cardiomyocytes were re‐introduced to the gradient concentration of calcium by stepwise adding of 0.2% (vol/vol) 100 mmol/L CaCl_2_, 0.5% (vol/vol) 100 mmol/L CaCl_2_ and 1% (vol/vol) 100 mmol/L CaCl_2_. After each time of calcium re‐introduction, the cell suspension was centrifuged at 20× *g* for 3 minutes. The final cell pellet was re‐suspended in myocyte plating medium (M199 culture medium, 10% FBS, 10mM BDM, 100 U/mL penicillin‐streptomycin, 2 mM ITS) and seeded in Matrigel‐coated cell culture plates. After 4 ~ 6 hours, the plating medium was changed to culture medium (M199 culture medium, 1 mg/mL BSA, 10 mM BDM, 100 U/mL penicillin‐streptomycin, 2 mM ITS).

### 2.5 Agilent extracellular seahorse analysis of glycolysis, glucose oxidation and fatty acid oxidation

Isolated cardiomyocytes were seeded on matrix gel–coated cell culture microplates (Agilent Seahorse XF24) at the cell intensity of 8000 cells/well. Generally, before performing the experiment, culture medium was changed into 500 μL assay medium (Agilent Seahorse XF Base Medium), and then, cells were incubated in 37°C non‐CO_2_ incubator for 1 hour.

*Glycolysis* Load 100mM glucose (56 μL), 10 μM oligomycin (62 μL) and 1 M 2‐deoxy‐glucose (2‐DG, 69 μL) into the corresponding injection port of sensor cartridge and do sensor cartridge calibration. Then, start the system to conduct basal extracellular acidification rate (ECAR) measurement [3 × (1.5 min mix, 2 min wait, 1.5 min measure)], which was followed by injection of glucose, oligomycin and 2‐DG successively, and each compound injection was followed by the ECAR measurement [3 × (1.5 min mix, 2 min wait, 1.5 min measure)].

*Glucose and pyruvate oxidation* Load 100 mM glucose (56 μL) or 10 mM pyruvate (56 μL) to injection port of sensor cartridge. After starting the measurement, the protocol consisted of basal oxygen consumption rate (OCR) measurement [3 × (1.5 min mix, 2 min wait, 1.5 min measure)] and fuel substrate–induced OCR measurement [3 × (1.5 min mix, 2 min wait, 1.5 min measure)] following glucose or pyruvate injection.

*Palmitate acid oxidation* Palmitate acid should be conjugated with BSA as previous report.^20^ 10mM palmitate‐BSA (56 μL) or BSA solution (56 μL) was loaded into ports of sensor cartridge and injected into the microplate following the basal OCR measurement [3 × (1.5 min mix, 2 min wait, 1.5 min measure)]. In addition, the palmitate acid–induced OCR measurement is 9 × (1.5 min mix, 2 min wait, 1.5 min measure).

### 2.6 Western blot

Protein extracted from rat heart tissue was separated by 8%‐12% SDS‐PAGE and then transferred to PVDF membrane for immunoblotting. The primary antibodies against P‐FOXO1 (S256), FOXO1, P‐PDH (S293), cleaved caspase 3, and histone 3 (H3) and GAPDH were purchased from Cell Signaling Technology, and PDK4 and CPT1 antibodies were purchased from Abcam. The intensity of protein bands was analysed by ImageJ software (National Institutes of Health).

### 2.7 Mitochondrial membrane potential detection by JC‐1 assay

The mitochondrial isolation from heart tissues was performed according to the manufacturer's instructions as per the Mitochondria Extraction Kit (Thermo Fisher Scientific). The isolated intact mitochondria were incubated with 2 μM JC‐1 stain in black 96‐well microplate for 10 minutes at 37°C. The fluorescent signal was determined by a fluorescence plate reader (Synergy HT BioTek) at excitation/emission of 485/535 nm for green fluorescence and 560/595 nm for red fluorescence.

### 2.8 Transmission electron microscopy of myocardium

The sample processing of fresh heart tissue for transmission electron microscopy study was according to the manual processing procedure issued by Electron Microscope Unit (The University of Hong Kong). The prepared slices were observed using Philips CM100 transmission electron microscopy.

### 2.9 Apoptotic cell death detection using terminal deoxynucleotidyl transferase dUTP nick‐end labelling

Terminal deoxynucleotidyl transferase dUTP nick‐end labelling (TUNEL) reaction was performed using an In Situ Cell Death Detection Kit (Roche Diagnostics GmbH) as previously described.^15^ The slides were observed on the microscope (Olympus BX41 fluorescence microscope) by an investigator who was initially blinded to treatment groups. The fluorescence intensity was analysed and quantified with ImageJ software (National Institutes of Health), and the apoptotic index was calculated as a percentage of staining‐positive nuclei to total nuclei.

### 2.10 Statistical analysis

All data were analysed by SPSS software version 19.0 (SPSS, Inc). One‐way analysis of variance (ANOVA) followed by multiple comparison Tukey test was used to compare the mean values among different experimental groups. All values are presented as means ± standard error of the mean (SEM). P value less than 0.05 was considered to indicate statistically significant differences.

## 3 RESULTS

### 3.1 AS1842856 treatment reduced myocardial FOXO1 nuclear translocation in diabetic hearts

**Figure 1:**
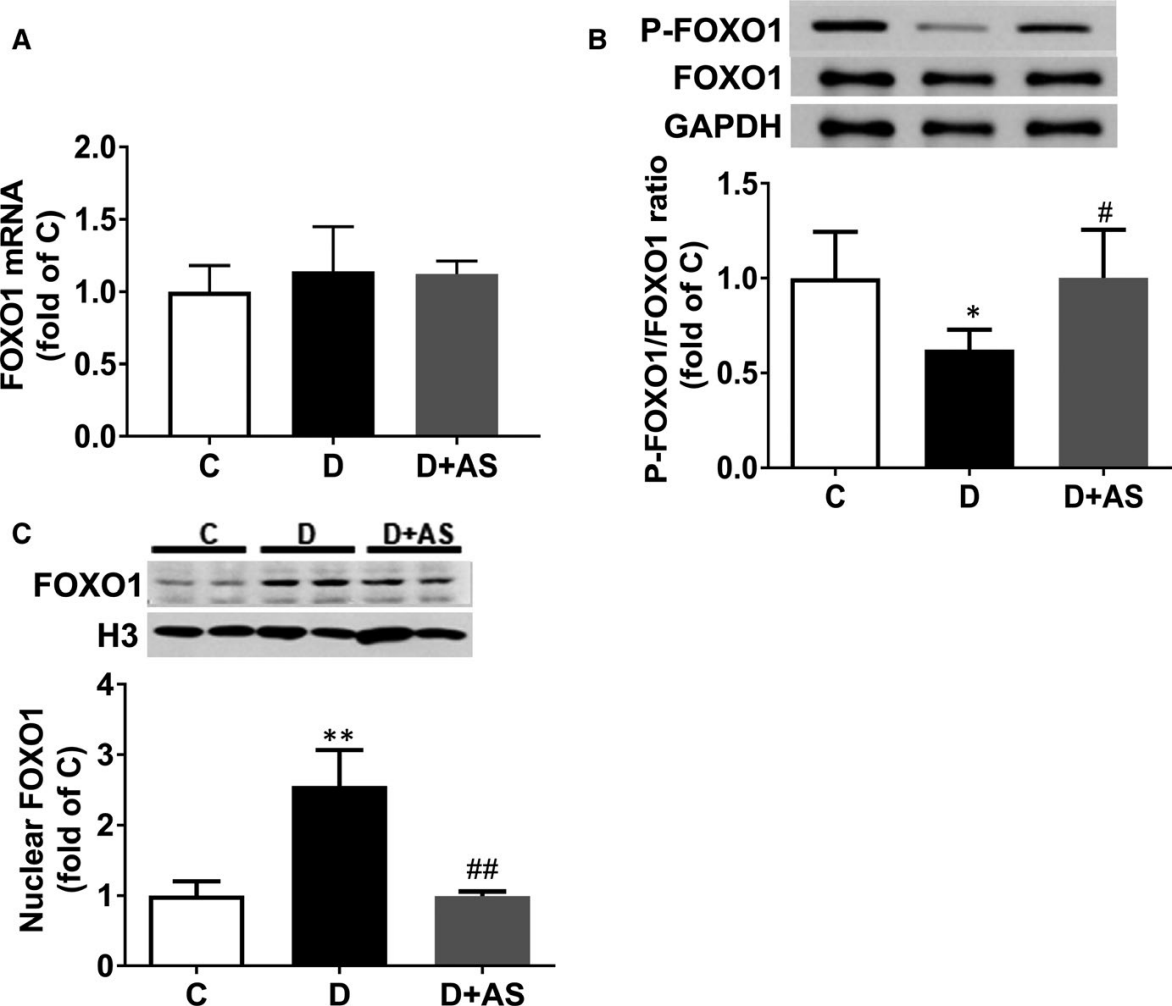
AS supplementation reduced myocardial P‐FOXO1/FOXO1 and nuclear presence of FOXO1 in 5‐wk diabetic rats. A, Cardiac mRNA level of FOXO1, B, P‐FOXO1/FOXO1 ratio and C, nuclear FOXO1 protein level were detected in non‐diabetes control (C), diabetic (D) and diabetic rats treated with AS (D + AS) groups. AS, AS1842856. Data are expressed as means ± SEM (n = 6) *P < .05 vs C; **P < .01 vs C; # *P* < .05 vs D.## *P* < .05 vs D

The phosphorylation of FOXO1 at the site ser256 enables the nuclear extrusion and inactivation of FOXO1 ^21^; thus, the protein ratio of P‐FOXO1 (S256)/FOXO1 can reflect the state of inactivation of FOXO1. As shown in Figure 1B, myocardial protein ratio of P‐FOXO1 (S256)/FOXO1 was significantly decreased in D rats at 5 weeks of the disease, whereas nuclear FOXO1 protein level (Figure 1C) was increased, indicating that FOXO1 is activated in diabetic hearts.

To investigate the role of FOXO1 in type 1 diabetes–induced DCM, FOXO1 antagonist AS was administered to STZ‐induced diabetic rats. Obviously, AS administration altered FOXO1 cellular location, manifested as significant increase in P‐FOXO1 (S256)/FOXO1 ratio and decrease in nuclear FOXO1 protein level (Figure 1B‐C). These results demonstrated that AS treatment prevented the increase in nuclear translocation of FOXO1 in diabetic myocardium, which contributed to FOXO1 inactivation.

### 3.2 AS1842856 treatment attenuated cardiac dysfunction in diabetic rats

Compared with non‐diabetic control (C) rats, D rats displayed polyphagia and morbid thirst, along with severe general abnormalities manifested as bodyweight loss, heart/body ratio increase, hyperglycaemia and hypertriglyceridaemia (Supplementary Table S1). AS treatment significantly reduced heart/body ratio and plasma triglyceride in D rats (Supplementary Table S1). PV loop analysis revealed reduced cardiac performance in D rats (Table 1), which was manifested as reduced HR, SV, SW, CO, LVESP, dP/dtmax and dP/dtmin. However, AS treatment significantly increased HR, CO and dP/dtmax (Table 1). These results indicated that treatment with AS improved clinically relevant abnormalities and cardiac function in D rats. These observations suggested that over‐activation of FOXO1 was involved in the pathology of DCM in type 1 diabetic rat.

**Table 1:** Haemodynamic variables and indices of cardiac systolic and diastolic function by a pressure‐volume conductance catheter system

Parameters	C	D	D + AS
HR (bpm)	422 ± 38	346 ± 15**	380 ± 22#
SV (μL)	191.92 ± 12.74	134.5 ± 23.83*	155.6 ± 19.72
SW (mm Hg/μL)	20 070 ± 363	9776 ± 1813**	11 357 ± 1125**
CO (mL/min)	85.34 ± 6.31	46.30 ± 6.07*	62.34 ± 2.59*#
LVESP (mm Hg)	136.35 ± 11.83	99.96 ± 9.54*	106.05 ± 18.76*
LVEDP (mm Hg)	3.53 ± 0.42	5.16 ± 0.50**	4.68 ± 0.52**
dP/dtmax (mm Hg/s)	9127 ± 1623	6479 ± 427*	9180 ± 465#
dP/dtmin (mm Hg/s)	10 179 ± 2310	6320 ± 351*	6941 ± 802*
Tau (ms)	7.78 ± 0.16	11.84 ± 1.29**	12.52 ± 0.31**

Note

All values are expressed as mean ± SEM. n = 6 per group. All these indices were measured among groups. *P < .05 vs C, **P < .01 vs C; #*P* < .05 vs D.

Abbreviations: AS, AS1842856; C, non‐diabetes control; CO, cardiac output; D + AS, diabetic rats treated with AS; D, diabetes; dP/dt max, maximal slope of systolic pressure increment; dP/dt min, diastolic decrement; HR, heart rate; LVEDP, left ventricular end‐diastolic pressure; LVESP, left ventricular end‐systolic pressure; SV, stroke volume; SW, stroke work; Tau, the relaxation time constant calculated by Weiss method.

### 3.3 AS1842856 treatment had no effects on impaired glycolysis in diabetic cardiomyocytes

**Figure 2:**
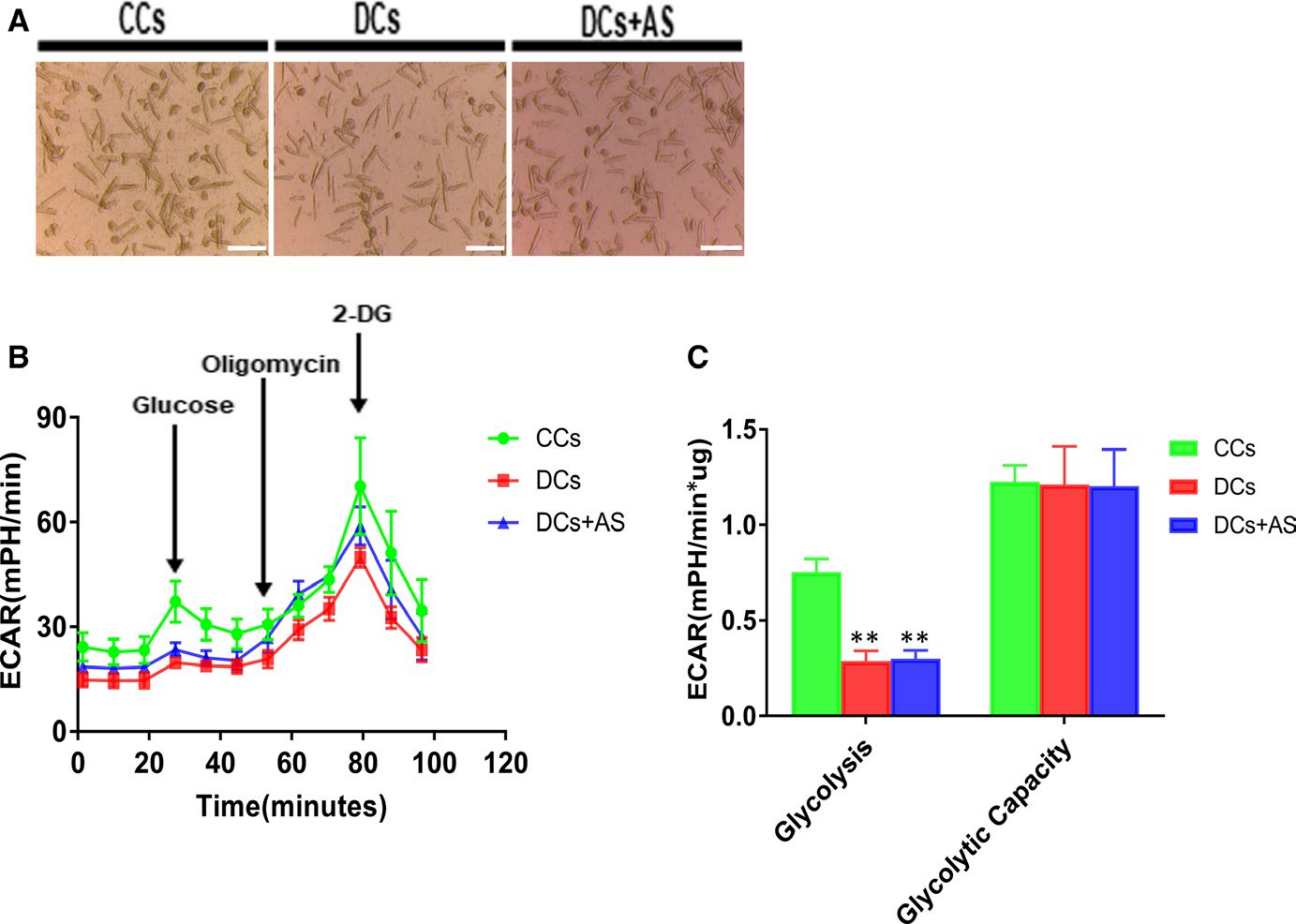
AS treatment did not affect glycolytic flux and glycolytic capacity in isolated diabetic cardiomyocytes. A, Isolated cardiomyocytes were seeded onto matrix gel–coated Seahorse XF24 Cell Culture Plates (8000 cells/well) treated with or without 1 μM AS for 24 h. Scale bar, 200 μm. B, Kinetic extracellular acidification rate (ECAR) responses of isolated cardiomyocytes to glucose (10 mM), oligomycin (1 μM) and 2‐DG (100 mM). C, Calculated glycolytic flux and glycolytic capacity. The former one is calculated by the ECAR increase (ECAR value of measurement 3 subtracted by measurement 4) normalized with cell protein content. The latter one is calculated by the ECAR increase (ECAR value of measurement 3 subtracted by measurement 9) normalized with cell protein content. Control cardiomyocytes (CCs); diabetic cardiomyocytes (DCs); AS‐treated diabetic cardiomyocytes (DCs + AS); AS, AS1842856; 2‐DG, 2‐deoxy‐glucose. Data are expressed as means ± SEM (n = 6). **P < .01 vs CCs

The isolated primary cardiomyocytes from C and D rats all displayed high viability with good morphology and connectivity (Figure 2A). To determine the effects of AS on glycolytic flux of these cardiomyocytes, the isolated cardiomyocytes were detected in DMEM assay medium following sequential addition of glucose, oligomycin (an inhibitor of ATP synthase) and 2‐deoxy‐glucose (2‐DG, an inhibitor of hexokinase). As illustrated in Figure 2B, glucose stimulated a large ECAR increase of 13.8 ± 4.4 mPH/min (difference between values of ECAR of measurement 3 and those of measurement 4) over basal glycolysis in isolated control cardiomyocytes (CCs), whereas glucose only evoked a slight ECAR increase in diabetic cardiomyocytes (DCs). After injection of oligomycin, cardiomyocytes in both CCs and DCs demonstrated the similar larger increase in ECAR, indicated as 20.2 ± 2.7 mPH/min (difference between values of ECAR of measurement 3 and those of measurement 9). The quantification as shown (Figure 2C) demonstrated that glycolysis was significantly decreased in DCs while the glycolytic capacity was basically unchanged as compared with CCs. Nevertheless, AS‐treated diabetic cardiomyocytes (DCs + AS) displayed similar bioenergetic profiles of glycolytic rate with DC group (Figure 2B and C). This indicated that AS treatment had no effects on impaired glycolytic rate in diabetic myocardium.

### 3.4 AS1842856 treatment reduced PDK4 expression and restored glucose oxidation in diabetic myocardium

**Figure 3:**
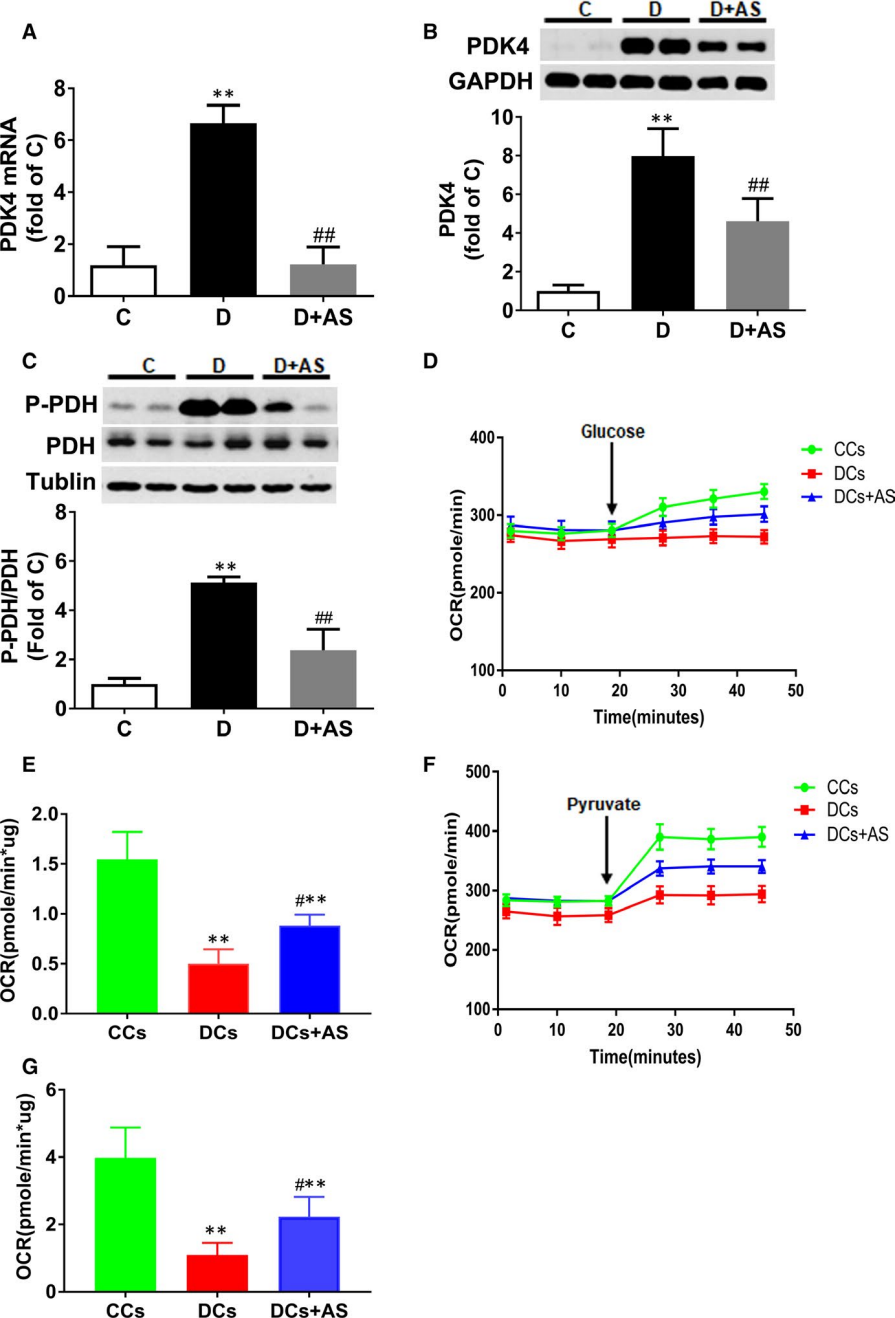
AS treatment reduced PDK4 and P‐PDH expression and restored glucose oxidation in diabetic myocardium. A‐C, Cardiac PDK4 mRNA level, PDK4 protein level and p‐PDH protein level. D, Kinetic oxygen consumption rate (OCR) responses of isolated cardiomyocytes to 10 mM glucose. E, Calculated glucose oxidation rate. F, Kinetic OCR responses of isolated cardiomyocytes to 1 mM pyruvate. G, Calculated pyruvate oxidation rate. The glucose or pyruvate oxidation rate was calculated by the OCR increase (OCR value of measurement 3 subtracted by measurement 6) normalized with cell protein content. AS, AS1842856. Data are expressed as means ± SEM (n = 6). *P < .05 vs C or CCs; **P < .01 vs C or CCs; #*P* < .05 vs D or DCs; ##*P* < .01 vs D or DCs

Pyruvate dehydrogenase (PDH) is a rate‐limiting enzyme that catalyses the conversion of pyruvate to acyl‐CoA and promotes glucose oxidation rate.^10^ PDK4 is the predominant isoform of PDKs in the heart, which can induce S256 phosphorylation of E1 subunit of PDH complex and whereby deactivate PDH.^22^ In the heart tissue from D rats, PDK4 mRNA and protein level were all significantly increased, respectively, demonstrated as ~ 6.7‐fold and ~7.97‐fold increase when compared with C group (Figure 3A and B), and p‐PDH was concomitantly enhanced (Figure 3C). However, AS treatment partially but significantly reduced myocardial PDK4 mRNA expression, PDK4 and P‐PDH in D rats.

Glucose addition triggered a significant OCR increase of 39.3 ± 6.9 pmole/min (difference between the value of OCR at measurement 3 and that at measurement 6) in CC group, but only induced a small increase of 4 ± 6.5 pmole/min in DCs (Figure 3D). Intriguingly, AS treatment enhanced the glucose‐stimulated OCR to 19.8 ± 5.9 pmole/min (difference between the value of OCR at measurement 3 and that at measurement 6) (Figure 3F). Figure 3E shows that glucose oxidation rate was impaired in diabetic myocardium (*P* < .01, CCs vs DCs) and AS treatment significantly restored it (*P* < .05, DCs + AS vs DCs). The result of pyruvate oxidation rate (Figure 3G) was similar to that of glucose oxidation rate. Taken together, these findings indicated that AS treatment could restore the reduced glucose oxidation and pyruvate oxidation rate in diabetic myocardium.

### 3.5 AS1842856 treatment significantly reduced the increase of fatty acid oxidation in diabetic myocardium

**Figure 4:**
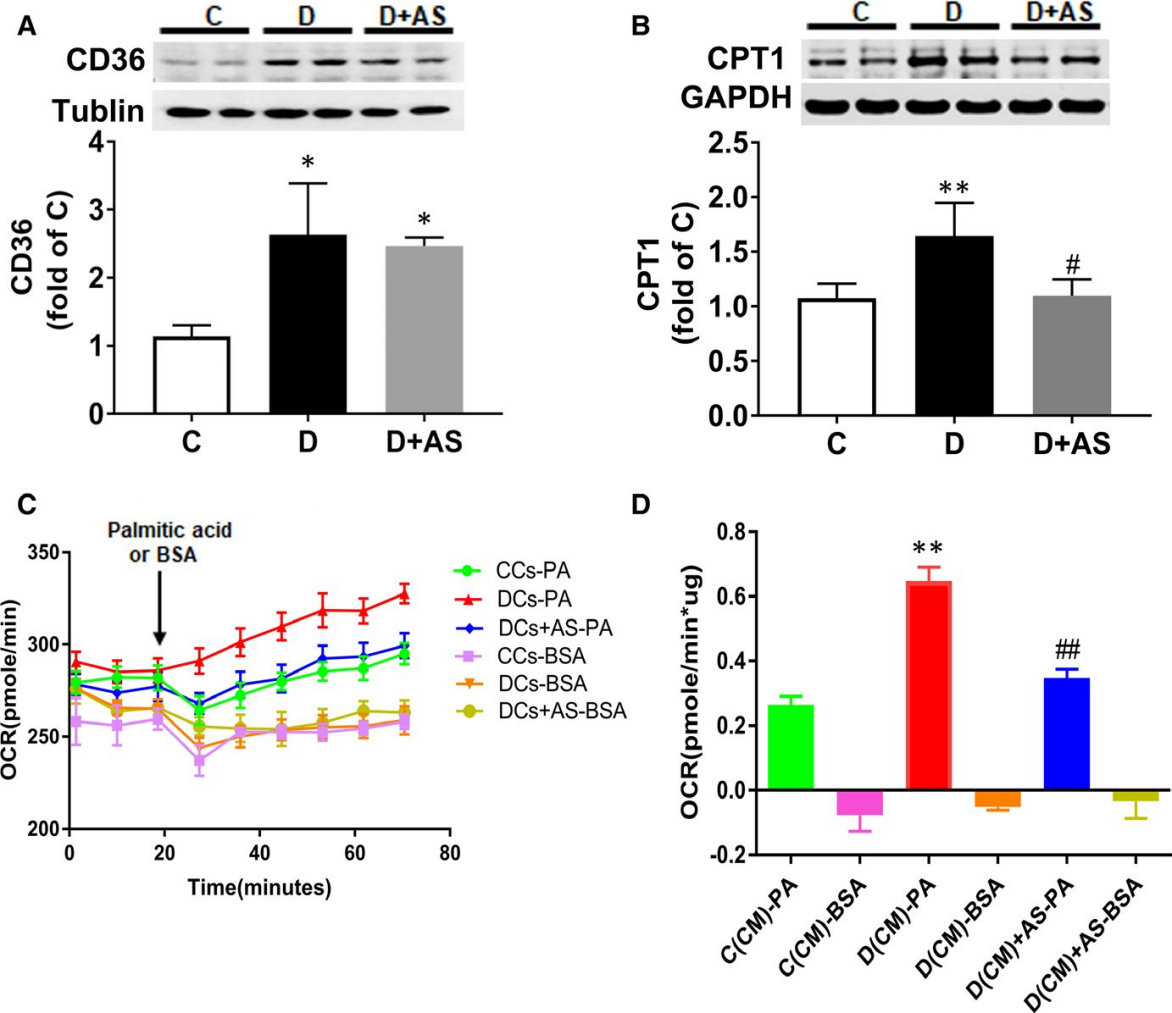
AS treatment significantly reduced diabetic myocardial CPT1 expression and concomitantly reduced increased palmitate oxidation in diabetic cardiomyocytes. A and B, Cardiac CD36 protein level and CPT1 protein level. C, Kinetic OCR responses of isolated cardiomyocytes to glucose 1 mM palmitate acid. D, Calculated palmitate acid oxidation. Palmitate acid oxidation is calculated by the OCR increase (OCR value of measurement 3 subtracted by measurement 9) normalized with cell protein content. OCR, oxygen consumption rate; AS, AS1842856. Data are expressed as means ± SEM (n = 6). *P < .05 vs C or CCs; **P < .01 vs C or CCs; #*P* < .05 vs D or DCs; ##*P* < .01 vs D or DCs

CD36, a fatty acid transporter, is responsible for primary fatty acid uptake in cardiac myocytes.^22^ Carnitine palmitoyltransferase 1 (CPT1) is a pivotal enzyme that controls long‐chain fatty acid access to the mitochondria for subsequent oxidative metabolism.^22^ These two enzymes are critical for fatty acid metabolism in the heart.^22^ In the present study, both CD36 and CPT1 were significantly elevated in diabetic hearts (Figure 4A and B), whereas AS treatment cancelled the increase in myocardial CPT1 expression without significant impact on CD36.

Palmitate acid was pre‐conjugated with BSA which was used as fatty acid oxidation substrates,^19^ and BSA alone was applied as vehicle control. Figure 4C illustrates that the addition of 1 mM palmitate‐BSA triggered a larger increase in OCR in DCs compared with CCs, manifested as 13 ± 2 vs 40.4 ± 5.3 pmole/min (CCs vs DCs, difference between value of OCR at measurement 3 and that at measurement 9), whereas AS treatment lowered down this increase to 22.5 ± 2.8 pmole/min (difference between value of OCR at measurement 3 and that at measurement 9). The quantification graph (Figure 4D) also revealed that DCs exhibited higher palmitate oxidation rate (*P* < .01, CCs vs DCs), which was abolished by AS treatment (*P* < .01, DCs + AS vs DCs).

### 3.6 AS1842856 treatment alleviated mitochondrial dysfunction and reduced apoptosis in diabetic hearts

**Figure 5:**
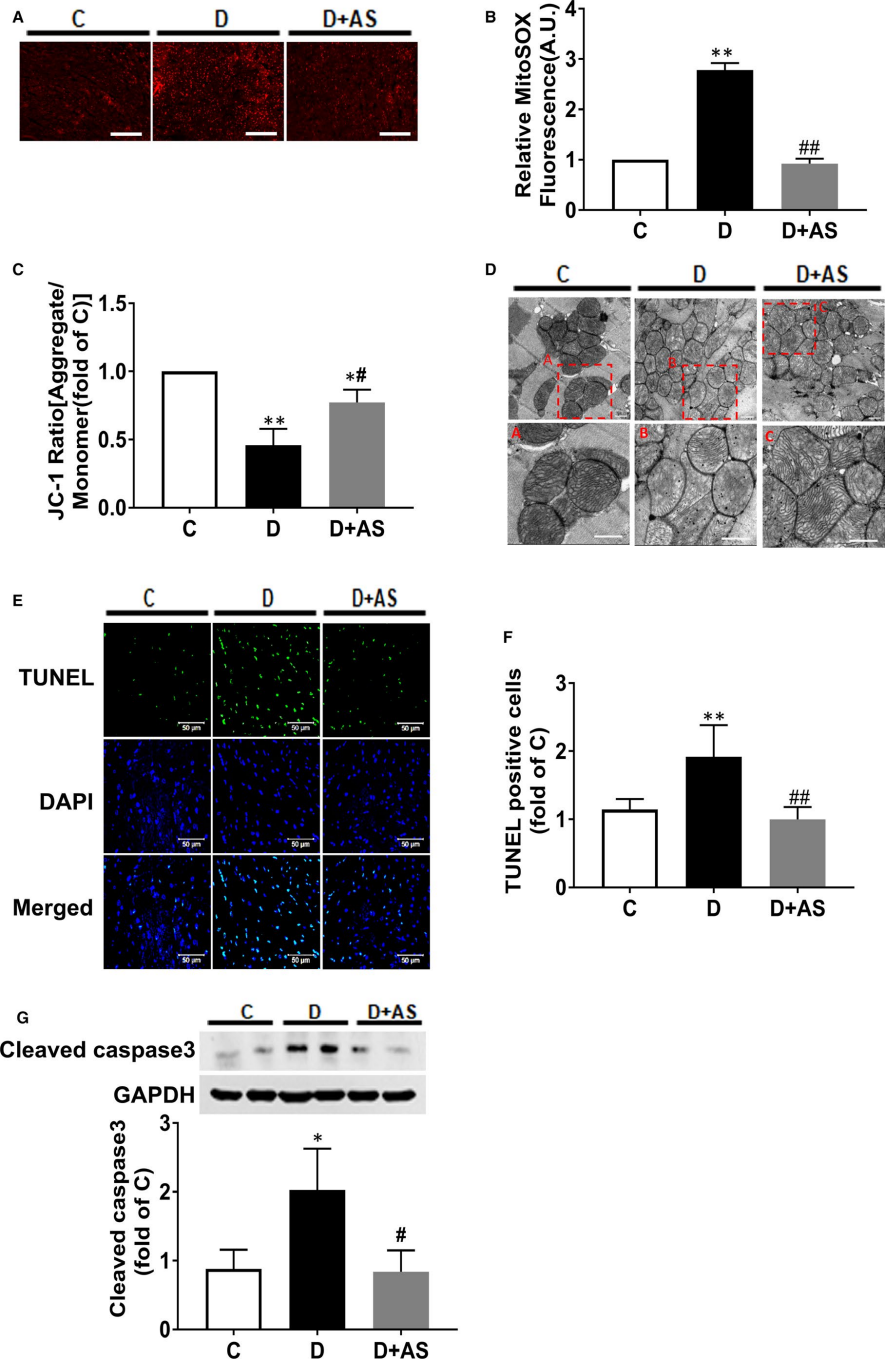
AS supplementation restored mitochondrial dysfunction and attenuated myocyte apoptosis in diabetic heart. A and B, mtROS was assessed by MitoSOX Red staining in frozen sections of heart tissues. Scale bar, 200 μM. C, Mitochondrial membrane potential by JC‐1 staining in mitochondria isolated from heart tissues. D, Representative electron photomicrographs of mitochondria (5200 × magnification) in heart tissue, and the given area (red rectangle) in original image (upper layer) was cropped and magnified (lower layer). Scale bar, 500 nM. E, F and G, Myocardial cell apoptosis assessed by cleaved caspase 3 protein expression and terminal deoxynucleotidyl transferase dUTP nick‐end labelling (TUNEL) assay. mtROS, mitochondrial reactive oxygen species; AS, AS1842856. Data are shown as means ± SEM, with n = 6 animals per group. *P < .05 vs C, **P < .01 vs C; # *P* < .05 vs D, ## *P* < .01 vs D

Diabetic hearts displayed excessive mtROS (Figure 5A and B), concomitant with decreased mitochondrial membrane potential (Figure 5C) and structural disorganization (Figure 5D). The morphology of mitochondria in diabetic heart was revealed by electron microscope as a loss of matrix density and disruption of inner membrane cristae (Figure 5D). Of note, AS administration remarkably reduced mtROS content and reverted mitochondrial membrane potential, as well as the morphology of mitochondrial structural, manifested by increased matrix density and organized cristae in inner membrane (Figure 5A‐D).

Diabetes significantly increased cardiac myocyte apoptosis, which was demonstrated as significantly increased TUNEL‐positive cells (Figure 5E and F) and cleaved caspase 3 protein level (Figure 5G). The above‐mentioned changes were all reverted by AS administration.

## 4 DISCUSSION

The myocardium is strong and capable of adjusting to fluctuations in circulating substrate concentrations,^23^ granting the heart the metabolic flexibility needed for feeding,^10^ fasting ^23^ and intense exercise.^24^ In diabetes, the cardiac energy flexibility (shift fuel preference) is constrained, which was demonstrated to be associated with aberrantly active myocardial FOXO1. In the present study, hearts from STZ‐induced diabetic rats exhibited increased FOXO1 nuclear translocation, PDK4/P‐PDH and CPT1 expression, concomitant with imbalanced oxidative metabolism, manifested by reduced glucose oxidation and elevated fatty acid oxidation. As anticipated, pharmacological inhibition of FOXO1 with AS significantly reduced the presence and/or activation of PDK4, PDH and CPT1, and concurrently normalized imbalanced oxidative metabolism. Moreover, AS treatment significantly attenuated the elevated mtROS, restored mitochondrial membrane potential (MMP) and mitochondrial structure and reduced apoptotic cells. Taken together, these results suggest that FOXO1, by regulating metabolic enzymes PDK4 and CPT1, is a key modulator of imbalanced oxidative metabolism in the myocardium of type 1 diabetic rats, and FOXO1 activation contributes to mitochondrial dysfunction and myocyte apoptosis, as well as cardiac dysfunction.

Hyperglycaemia induces various adverse effects on heart, including glucotoxicity and over‐production of advanced glycation end products, which contribute to myocardial fibrosis, cardiac stiffness and impaired diastolic relaxation in diabetes.^25^ In 2010, Nagashima et al firstly reported that AS can bind to Foxo1 via mass spectrometric affinity screening analysis and inhibit FOXO1‐mediated transactivation, indicating that AS works as a selective inhibitor of FOXO1.^26^ Acute administration of AS in diabetic db/db mice has been shown to significantly decrease liver gluconeogenesis and thus decrease fasting plasma glucose.^26^ In the current study, AS treatment reduced mitochondrial ROS content and cardiac apoptosis and improved cardiac function, but did not have any significant effects on existing hyperglycaemia in STZ‐induced diabetic rats (Supplementary Table S1). This result may suggest that the beneficial effects of AS treatment on DCM in T1DM are not through lowering blood glucose.

In diabetic hearts, the compromised glucose metabolism is manifested by impaired glycolysis and decreased glucose oxidation.^27^ The role of activated FOXO1 in impaired glycolysis and reduced glucose oxidation in diabetic hearts in T1DM has not yet been assessed before. Pyruvate, as the end product of glycolysis, can be directly consumed by mitochondria ^28^ and thus could skip the step of glucose uptake. Our results showed AS treatment did not alter the impaired glycolytic flux but could induce pronounced increase in pyruvate oxidation. Moreover, the restored extent of pyruvate oxidation (~2‐fold increase) (Figure 3G) in diabetic myocardium after AS treatment was comparable to that of glucose oxidation (~1.8‐fold increase) (Figure 3E), suggesting that AS treatment merely restored glucose oxidation process alone rather than affecting the processes of glucose uptake and glycolysis.

PDH converts pyruvate to acetyl‐CoA, linking glycolysis to the Krebs cycle, and plays an important role in glucose metabolism in cardiac myocytes.^10^, ^29^ PDK4, a dominant isoform of PDKs in the heart, functions to phosphorylate and inactivate PDH and reduces its capacity to oxidize glucose.^30^, ^31^ Recently, FOXO1 has been recognized as a novel upstream regulator of PDK4 and FOXO1 activation confines glucose availability for oxidation in cardiac myocytes.^10^ However, the direct link among FOXO1, PDK4 and glucose oxidation in diabetic heart has not yet been documented. Our study showed that diabetic myocardium displayed increased FOXO1 nuclear translocation, and elevated PDK4 and P‐PDH expression, which were concomitant with significantly decreased glucose oxidation rate (Figure 3D and E). However, AS treatment reversed the above alterations (Figure 3D and E). This result suggested that aberrantly active FOXO1 inhibited glucose oxidation via enhancing PDK4 expression. Unlike the total cancellation of mRNA expression, AS treatment induced approximately 50% reduction in PDK4 protein level in hearts from D rats (Figure 3B). A possible reason might be that degradation rate of PDK4 in diabetes is decayed due to the increased availability of acetyl‐CoA.^32^, ^33^ The declined degradation made PDK4 content still at high level although AS treatment already reduced its mRNA to normal level. In addition, the incomplete inhibition of PDK4 level may serve to support the notion that AS1842956 treatment could not completely reduce P‐PDH level and restore glucose oxidation.

Increased circulating triglyceride can augment cardiac fatty acid uptake and lead to lipotoxicity in cardiomyocytes.^22^ Likewise, increased plasma triglyceride was observed in STZ‐induced insulin‐deficient rats (Supplementary Table S1). Until now, the underlying mechanism regarding how insulin deficiency causes hypertriglyceridaemia is still unclear. In the present study, AS treatment reduced diabetic plasma triglyceride, implicating that FOXO1 may contribute to hypertriglyceridaemia in T1DM. Altomonte et al^34^ have shown that FOXO1 can stimulate the expression of apolipoprotein CIII in the liver, which can inhibit peripheral clearance of VLDL triglycerides. Intriguingly, activated FOXO1 has been demonstrated in the liver of STZ‐induced D rats in our recent study.^35^ However, whether activated FOXO1 in the liver augments the apolipoprotein expression and thus contributes to hypertriglyceridaemia in T1DM merits further investigation. In addition, enhanced myocardial fatty acid uptake has been demonstrated in both T1DM and T2DM animal models,^36^, ^37^ which was associated with enhanced cardiac fatty acid transporter CD36 expression. In a previous study, cardiac‐specific knockout of FOXO1 cancelled the increase in CD36 mRNA expression and reduced lipotoxicity in the hearts of T2DM mice.^12^ In our current study, heart from STZ‐induced T1DM rats displayed elevated CD36 mRNA expression, but inhibition of FOXO1 by AS had no effect on increased CD36 mRNA expression (Figure 4). Although several other reports indicate that FOXO1 regulates CD36 sarcolemmal membrane translocation without affecting its transcriptional expression,^11^ here we focused on the effects of FOXO1 on fatty acid oxidation rather than fatty acid uptake.

Excessive fatty acid oxidation leads to cardiac inefficiency ^36^ and mitochondrial injury,^7^ all of which contribute to the abnormalities in cardiac function observed in DCM. In the present study, diabetic hearts demonstrated elevated fatty acid oxidation. This was associated with enhanced transcript levels for enzyme that catalyse fatty acid oxidation, CPT1. CPT1 controls fatty acid access to the mitochondria, and thus, it is deemed to play critical roles in aberrantly elevated mitochondrial fatty acid oxidation in the setting of diabetic hearts.^33^ For instance, CPT‐1 inhibitors (*eg* perhexiline and etomoxir), which target mitochondrial fatty acid oxidation, have been proved to be beneficial to diabetic heart.^37^ Likewise, in our current study, pharmacological inhibition of FOXO1 significantly reduced the increases of CPT1 and fatty acid oxidation (Figure 4). This suggests that FOXO1 activation enhances CPT1, which further augments fatty acid oxidation in diabetic heart. To our knowledge, CPT1 expression has been reported to be under the transcriptional regulation of PPARα in the liver.^38^ But, this transcription regulatory relationship has not been verified in the heart. Especially, a decrease in cardiac PPARα ^39^, ^40^ and an increase in CPT1 are observed in STZ‐induced diabetes,^39^ suggesting that there exist other unknown signalling pathways in the regulation of CPT1 expression in diabetic hearts. Our finding that treatment with AS decreased CPT1 expression implicates that FOXO1 may serve as a potential upstream regulator of CPT1, which provides a novel mechanism for the regulation of this enzyme in diabetic heart.

Mitochondria are vulnerable to altered energy metabolism and the functional or morphological pathological changes of which lead to cardiac dysfunction, and this is accompanied by increased cardiomyocyte apoptosis.^33^ As mentioned above, diabetic hearts rely more on mitochondrial fatty acid oxidation for its ATP requirements. However, the abnormal increment of fatty acid metabolism may be energetically detrimental to the mitochondria, as there will be higher delivery of FADH_2_ and NADH to the electronic transport chain ^14^ and more oxygen consumption when oxidizing more fatty acids, leading to enhanced mtROS generation.^33^ Then, the excessive mtROS production triggers mitochondrial oxidative stress, leads to mitochondrial respiratory uncoupling and MMP depolarization ^41^ and further results in mitochondrial dysfunction and cell apoptosis. Similarly, in the present study, diabetic heart tissue demonstrated excessive mtROS, depolarized MMP and disrupted mitochondrial structure, which were concomitant with increased apoptotic cardiac myocytes (Figure 5). Pharmacological inhibition of FOXO1 not only restored the balance of oxidative metabolism and especially prevented the elevation of fatty acid oxidation in diabetic cardiomyocytes but also reversed mitochondrial dysfunction, concomitant with reduction of mtROS and restoration of MMP, and attenuation of myocyte apoptosis (Figure 5). Hence, we postulated that FOXO1‐induced fatty acid oxidation augmentation contributes to the excessive mtROS production in diabetic heart. The increased mtROS is then attributable to the loss of MMP and subsequent cardiomyocyte apoptosis. The fact that mitochondrial dysfunction, cell apoptosis and cardiac dysfunction were all improved following the restoration of oxidative metabolism in diabetic heart by AS treatment indicates an important role of FOXO1 over‐activation in this pathology.

It should be noted that AS (FOXO1 inhibitor) treatment, rather than cardiac‐specific FOXO1 knockout mice, was used to study the cardiometabolic role of FOXO1 in type 1 DCM. The present study may not define a definite role of FOXO1 in the development of DCM in T1DM. However, it should be noted that FOXO1 plays an essential role in sustaining cardiomyocyte metabolism and cell survival.^9^ Indeed, in mice with cardiac‐specific deficiency of FOXO1, the heart demonstrated lowered systole,^42^ potentially arrhythmias,^43^ when compared to wild‐type mice. Hence, the moderate amount of FOXO1 is required to maintain its physiological function. Thus, AS administration makes it possible to inhibit excessive FOXO1 activity in diseased models and the current experimental setting provide a more physiological condition instead of total abrogation of FOXO1 gene.

## 5 CONCLUSIONS

**Figure 6:**
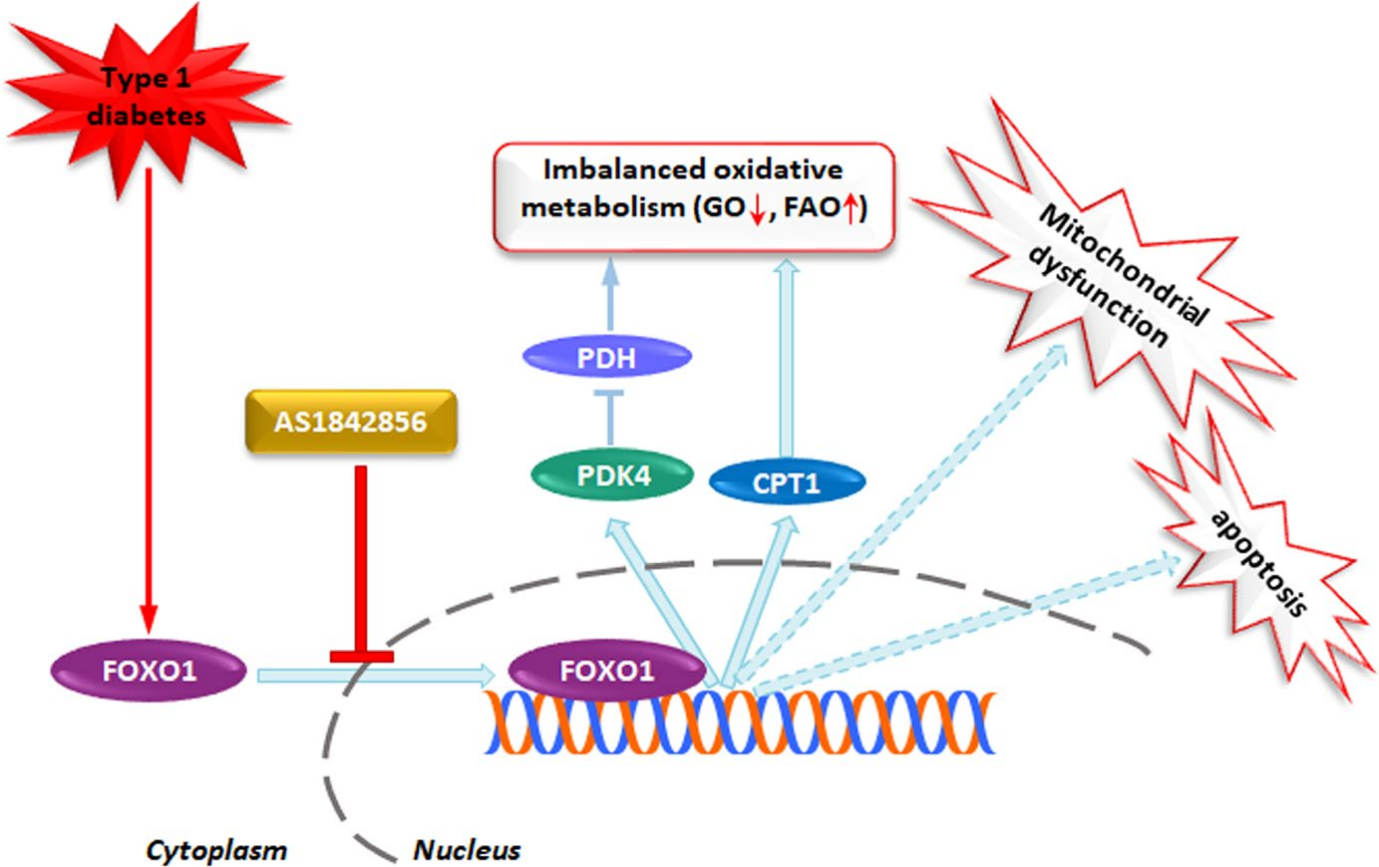
Schematic illustration of proposed signalling mechanism involved in diabetes‐induced FOXO1 activation promotes imbalanced oxidative metabolism, mitochondrial dysfunction and cardiac dysfunction in heart. Under the condition of diabetes, FOXO1, by enhancing PDK4 and CPT1 expression, induces imbalanced oxidative metabolism manifested as decreased glucose oxidation (GO) and elevated fatty acid oxidation (FAO), which was concomitant with mitochondrial dysfunction and cardiac dysfunction. However, AS1842856 treatment attenuated or prevented all the changes via inhibition of diabetes‐induced FOXO1 nuclear translocation

To summarize, over‐activation of FOXO1, through increasing PDK4 and CPT1 expression, induced disarranged cardiac oxidative metabolism, manifested by a shift in substrate preference from glucose oxidation to fatty acid oxidation, and subsequently caused mitochondrial dysfunction and cardiac myocyte apoptosis, thus leading to cardiac dysfunction in STZ‐induced diabetic rat (Figure 6). Most of all, AS treatment reverted all above‐mentioned alterations and resulted in the alleviation of DCM. Data reported here suggest that targeting FOXO1 by AS, by restoring balanced mitochondrial oxidative metabolism, may have the potential to be a promising treatment for heart failure and DCM.

## CONFLICT OF INTEREST

The authors confirm that there are no conflicts of interest regarding the publication of this article.

## AUTHOR CONTRIBUTIONS

DY performed the research and wrote the manuscript. YC designed the research study and analysed the data. JL, JL, XL, FY and XX performed the study. X. L and AX contributed to data analysis and interpretation. XM reviewed and approved the research protocol. ZX reviewed and approved the research protocol and wrote the manuscript. ZX is the guarantor of this work and, as such, had full access to all the data in the study and took responsibility for the integrity of the data and the accuracy of the data analysis.

## Supporting information

Table S1

## Data Availability

The data sets used and/or analysed during the current study are available from the corresponding author on reasonable request.

## References

[1] Jia G , Hill MA , Sowers JR . Diabetic cardiomyopathy: an update of mechanisms contributing to this clinical entity. Circ Res. 2018;122(4):624‐638. 29449364 10.1161/CIRCRESAHA.117.311586 PMC5819359

[2] Varma U , Koutsifeli P , Benson VL , et al. Molecular mechanisms of cardiac pathology in diabetes ‐ Experimental insights. Biochim Biophys Acta‐Mol Basis Dis. 2018;1864(5):1949‐1959. 29109032 10.1016/j.bbadis.2017.10.035

[3] Holscher ME , Bode C , Bugger H . Diabetic cardiomyopathy: does the type of diabetes matter? Int J Mol Sci. 2016;17(12):2136. 10.3390/ijms17122136 PMC5187936 27999359

[4] De Blasio MJ , Huynh N , Deo M , et al. Defining the progression of diabetic cardiomyopathy in a mouse model of type 1 diabetes. Front Physiol. 2020;11:124. 32153425 10.3389/fphys.2020.00124 PMC7045054

[5] Herrero P , Peterson LR , McGill JB , et al. Increased myocardial fatty acid metabolism in patients with type 1 diabetes mellitus. J Am Coll Cardiol. 2006;47(3):598‐604. 16458143 10.1016/j.jacc.2005.09.030

[6] Bayeva M , Sawicki KT , Ardehali H . Taking diabetes to heart–deregulation of myocardial lipid metabolism in diabetic cardiomyopathy. J Am Heart Assoc. 2013;2(6):e000433. 24275630 10.1161/JAHA.113.000433 PMC3886738

[7] Verma SK , Garikipati VNS , Kishore R . Mitochondrial dysfunction and its impact on diabetic heart. Biochim Biophys Acta‐Mol Basis Dis. 2017;1863(5):1098‐1105. 27593695 10.1016/j.bbadis.2016.08.021 PMC5332436

[8] Kousteni S . FoxO1, the transcriptional chief of staff of energy metabolism. Bone. 2012;50(2):437‐443. 21816244 10.1016/j.bone.2011.06.034 PMC3228887

[9] Puthanveetil P , Wan A , Rodrigues B . FoxO1 is crucial for sustaining cardiomyocyte metabolism and cell survival. Cardiovasc Res. 2013;97(3):393‐403. 23263330 10.1093/cvr/cvs426

[10] Gopal K , Saleme B , Al Batran R , et al. FoxO1 regulates myocardial glucose oxidation rates via transcriptional control of pyruvate dehydrogenase kinase 4 expression. Am J Physiol Heart Circ Physiol. 2017;313(3):H479‐H490. 28687587 10.1152/ajpheart.00191.2017

[11] Puthanveetil P , Wang Y , Zhang D , et al. Cardiac triglyceride accumulation following acute lipid excess occurs through activation of a FoxO1‐iNOS‐CD36 pathway. Free Radic Biol Med. 2011;51(2):352‐363. 21545834 10.1016/j.freeradbiomed.2011.04.009

[12] Battiprolu PK , Hojayev B , Jiang N , et al. Metabolic stress‐induced activation of FoxO1 triggers diabetic cardiomyopathy in mice. J Clin Invest. 2012;122(3):1109‐1118. 22326951 10.1172/JCI60329 PMC3287230

[13] Piao L , Sidhu VK , Fang YH , et al. FOXO1‐mediated upregulation of pyruvate dehydrogenase kinase‐4 (PDK4) decreases glucose oxidation and impairs right ventricular function in pulmonary hypertension: therapeutic benefits of dichloroacetate. J Mol Med (Berl). 2013;91(3):333‐346. 23247844 10.1007/s00109-012-0982-0 PMC3584201

[14] Lorenzo O , Ramirez E , Picatoste B , et al. Alteration of energy substrates and ROS production in diabetic cardiomyopathy. Mediators Inflamm. 2013;2013:461967. 24288443 10.1155/2013/461967 PMC3833358

[15] Luo J , Yan D , Li S , et al. Allopurinol reduces oxidative stress and activates Nrf2/p62 to attenuate diabetic cardiomyopathy in rats. J Cell Mol Med. 2020;24(2):1760‐1773. 31856386 10.1111/jcmm.14870 PMC6991641

[16] Li H , Yao W , Irwin MG , et al. Adiponectin ameliorates hyperglycemia‐induced cardiac hypertrophy and dysfunction by concomitantly activating Nrf2 and Brg1. Free Radic Biol Med. 2015;84:311‐321. 25795513 10.1016/j.freeradbiomed.2015.03.007

[17] Li H , Liu Z , Wang J , et al. Susceptibility to myocardial ischemia reperfusion injury at early stage of type 1 diabetes in rats. Cardiovasc Diabetol. 2013;12:133. 24041262 10.1186/1475-2840-12-133 PMC3847499

[18] Xu JJ , Li HB , Irwin MG , et al. Propofol ameliorates hyperglycemia‐induced cardiac hypertrophy and dysfunction via heme oxygenase‐1/signal transducer and activator of transcription 3 signaling pathway in rats. Crit Care Med. 2014;42(8):E583‐E594. 24810525 10.1097/CCM.0000000000000415

[19] Lei S , Li H , Xu J , et al. Hyperglycemia‐induced protein kinase C beta2 activation induces diastolic cardiac dysfunction in diabetic rats by impairing caveolin‐3 expression and Akt/eNOS signaling. Diabetes. 2013;62(7):2318‐2328. 23474486 10.2337/db12-1391 PMC3712061

[20] Wang D , Green MF , McDonnell E , Hirschey MD . Oxygen flux analysis to understand the biological function of sirtuins. Methods Mol Biol. 2013;1077:241‐258. 24014411 10.1007/978-1-62703-637-5_16 PMC3817486

[21] Tzivion G , Dobson M , Ramakrishnan G . FoxO transcription factors; regulation by AKT and 14‐3‐3 proteins. Biochim Biophys Acta. 2011;1813(11):1938‐1945. 21708191 10.1016/j.bbamcr.2011.06.002

[22] Lopaschuk GD , Ussher JR , Folmes CD , et al. Myocardial fatty acid metabolism in health and disease. Physiol Rev. 2010;90(1):207‐258. 20086077 10.1152/physrev.00015.2009

[23] Griffin TM , Humphries KM , Kinter M , et al. Nutrient sensing and utilization: getting to the heart of metabolic flexibility. Biochimie. 2016;124:74‐83. 26476002 10.1016/j.biochi.2015.10.013 PMC4828282

[24] Gibb AA , Epstein PN , Uchida S , et al. Exercise‐induced changes in glucose metabolism promote physiological cardiac growth. Circulation. 2017;136(22):2144‐2157. 28860122 10.1161/CIRCULATIONAHA.117.028274 PMC5704654

[25] Jia GH , Whaley‐Connell A , Sowers JR . Diabetic cardiomyopathy: a hyperglycaemia‐ and insulin‐resistance‐induced heart disease. Diabetologia. 2018;61(1):21‐28. 28776083 10.1007/s00125-017-4390-4 PMC5720913

[26] Nagashima T , Shigematsu N , Maruki R , et al. Discovery of novel forkhead box O1 inhibitors for treating type 2 diabetes: improvement of fasting glycemia in diabetic db/db mice. Mol Pharmacol. 2010;78(5):961‐970. 20736318 10.1124/mol.110.065714

[27] Stanley WC , Lopaschuk GD , McCormack JG . Regulation of energy substrate metabolism in the diabetic heart. Cardiovasc Res. 1997;34(1):25‐33. 9217869 10.1016/s0008-6363(97)00047-3

[28] Readnower RD , Brainard RE , Hill BG , Jones SP . Standardized bioenergetic profiling of adult mouse cardiomyocytes. Physiol Genomics. 2012;44(24):1208‐1213. 23092951 10.1152/physiolgenomics.00129.2012 PMC3544486

[29] Patel MS , Nemeria NS , Furey W , Jordan F . The pyruvate dehydrogenase complexes: structure‐based function and regulation. J Biol Chem. 2014;289(24):16615‐16623. 24798336 10.1074/jbc.R114.563148 PMC4059105

[30] Wu PF , Sato J , Zhao Y , et al. Starvation and diabetes increase the amount of pyruvate dehydrogenase kinase isoenzyme 4 in rat heart. Biochem J. 1998;329:197‐201. 9405294 10.1042/bj3290197 PMC1219032

[31] Zhao G , Jeoung NH , Burgess SC , et al. Overexpression of pyruvate dehydrogenase kinase 4 in heart perturbs metabolism and exacerbates calcineurin‐induced cardiomyopathy. Am J Physiol Heart Circ Physiol. 2008;294(2):H936‐H943. 18083902 10.1152/ajpheart.00870.2007

[32] Schafer C , Young ZT , Makarewich CA , et al. Coenzyme A‐mediated degradation of pyruvate dehydrogenase kinase 4 promotes cardiac metabolic flexibility after high‐fat feeding in mice. J Biol Chem. 2018;293(18):6915‐6924. 29540486 10.1074/jbc.RA117.000268 PMC5936801

[33] Heather LC , Clarke K . Metabolism, hypoxia and the diabetic heart. J Mol Cell Cardiol. 2011;50(4):598‐605. 21262230 10.1016/j.yjmcc.2011.01.007

[34] Altomonte J , Cong L , Harbaran S , et al. Foxo1 mediates insulin action on apoC‐III and triglyceride metabolism. J Clin Invest. 2004;114(10):1493‐1503. 15546000 10.1172/JCI19992 PMC525736

[35] Xie X , Yan D , Li HB , et al. Enhancement of adiponectin ameliorates nonalcoholic fatty liver disease via inhibition of FOXO1 in type I diabetic rats. J Diabetes Res. 2018;9. 10.1155/2018/6254340 PMC6116459 30186875

[36] Fillmore N , Lopaschuk GD . Targeting mitochondrial oxidative metabolism as an approach to treat heart failure. Biochim Biophys Acta. 2013;1833(4):857‐865. 22960640 10.1016/j.bbamcr.2012.08.014

[37] Rupp H , Zarain‐Herzberg A , Maisch B . The use of partial fatty acid oxidation inhibitors for metabolic therapy of angina pectoris and heart failure. Herz. 2002;27(7):621‐636. 12439634 10.1007/s00059-002-2428-x

[38] Song S , Attia RR , Connaughton S , et al. Peroxisome proliferator activated receptor alpha (PPARalpha) and PPAR gamma coactivator (PGC‐1alpha) induce carnitine palmitoyltransferase IA (CPT‐1A) via independent gene elements. Mol Cell Endocrinol. 2010;325(1–2):54‐63. 20638986 10.1016/j.mce.2010.05.019 PMC3160239

[39] Lee T‐I , Kao Y‐H , Chen Y‐C , et al. Cardiac metabolism, inflammation, and peroxisome proliferator‐activated receptors modulated by 1,25‐dihydroxyvitamin D3 in diabetic rats. Int J Cardiol. 2014;176(1):151‐157. 25062566 10.1016/j.ijcard.2014.07.021

[40] Lee TW , Bai KJ , Lee TI , et al. PPARs modulate cardiac metabolism and mitochondrial function in diabetes. J Biomed Sci. 2017;24(1):5. 28069019 10.1186/s12929-016-0309-5 PMC5223385

[41] Johnson R , Dludla P , Joubert E , et al. Aspalathin, a dihydrochalcone C‐glucoside, protects H9c2 cardiomyocytes against high glucose induced shifts in substrate preference and apoptosis. Mol Nutr Food Res. 2016;60(4):922‐934. 26773306 10.1002/mnfr.201500656

[42] Hu XL , Xu X , Huang YM , et al. Disruption of sarcolemmal ATP‐sensitive potassium channel activity impairs the cardiac response to systolic overload. Circ Res. 2008;103(9):1009‐U222. 18802029 10.1161/CIRCRESAHA.107.170795 PMC2877276

[43] Cai BZ , Wang N , Mao WK , et al. Deletion of FoxO1 leads to shortening of QRS by increasing Na+ channel activity through enhanced expression of both cardiac Na(v)1.5 and beta 3 subunit. J Mol Cell Cardiol. 2014;74:297‐306. 24956219 10.1016/j.yjmcc.2014.06.006 PMC4158923

