# Supplementary material for: FOXO1 contributes to diabetic cardiomyopathy via inducing imbalanced oxidative metabolism in type 1 diabetes
Source: J Cell Mol Med. 2020 May 25;24(14):7850–61. doi: 10.1111/jcmm.15418 (PMC7348139; doi:10.1111/jcmm.15418)
Supplement: Supplementary file 1 — Table S1 [file JCMM-24-7850-s001.docx]

**Supplementary Table**

Table S1. General characteristics after AS supplementation at termination of study.

| Parameters | C | D | D+ AS |
| --- | --- | --- | --- |
| Food intake(g/kg/day) | 76.5±1.7 | 186.6±21.4** | 169.2±22.3** |
| Water consumption  (ml/kg/day) | 114.3±9.4 | 897.0±108.5** | 937.7±161.9** |
| Heart/Body ratio (g/kg) | 2.44±0.12 | 3.06±0.32** | 2.52±0.08## |
| Blood glucose (mM) | 7.6±06 | 31.5±2.6** | 32.4±1.8** |
| Plasma triglyceride (nM) | 813.9±99.5 | 2059.3±105** | 883.6±305.5## |

Food intake and water consumption were the average value of the last week of the study when 50mg/kg AS was administered to the subgroup of diabetic rats. Body weight, plasma glucose and triglyceride were determined at the end of the experiment. C, Non-diabetes control; D, diabetes; D+AS, diabetic rats treated with AS; AS, AS1842856. All values are expressed as mean ± S.E.M. n=6 per group. **, p<0.01 *vs*. C, ##, p<0.01 *vs*. D.
